# Proposal for an Update of the Definition and Scope of Behavioral Medicine

**DOI:** 10.1007/s12529-016-9610-7

**Published:** 2016-11-14

**Authors:** Joost Dekker, Adrienne Stauder, Frank J. Penedo

**Affiliations:** 1 Department of Psychiatry and Department of Rehabilitation Medicine, VU University Medical Center, PO Box 7057, 1007 MB Amsterdam, the Netherlands; 2 Jining Medical University, Jining, China; 3 Faculty of Sports and Health Sciences, University of Jyväskylä, Jyväskylä, Finland; 4 Institute of Behavioral Sciences, Semmelweis University Budapest, Budapest, Hungary; 5 Departments of Medical Social Sciences, Psychology and Psychiatry and Behavioral Sciences, Northwestern University, Chicago, USA

**Keywords:** Behavioral medicine, Definition, Scope

## Abstract

**Purpose:**

We aim to provide an update of the definition and scope of behavioral medicine in the Charter of ISBM, as the present version was developed more than 25 years ago.

**Methods:**

We identify issues which need clarification or updating. This leads us to propose an update of the definition and scope of behavioral medicine.

**Results:**

Issues in need of clarification or updating include the scope of behavioral medicine (biobehavioral mechanisms, clinical diagnosis and intervention, and prevention and health promotion); research as an essential characteristic of all three areas of behavioral medicine; the application of behavioral medicine; the terminology of behavioral medicine as a multidisciplinary field; and the relationship and distinction between behavioral medicine, mental health, health psychology, and psychosomatic medicine.

**Conclusion:**

We propose the following updated definition and scope of behavioral medicine: “Behavioral medicine can be defined as the multidisciplinary field concerned with the development and integration of biomedical and behavioral knowledge relevant to health and disease, and the application of this knowledge to prevention, health promotion, diagnosis, treatment, rehabilitation, and care. The scope of behavioral medicine extends from biobehavioral mechanisms (i.e., the interaction of biomedical processes with psychological, social, societal, cultural, and environmental processes), to clinical diagnosis and intervention, and to public health.”

## Introduction

Behavioral medicine was defined in the Charter of the International Society of Behavioral Medicine (ISBM) more than 25 years ago. The original definition of the field behavioral medicine was developed at the Yale Conference on behavioral medicine and later published by Schwartz and Weiss [1, 2]. The definition in the ISBM Charter is a refinement of that definition. The field of behavioral medicine has significantly evolved and advanced over the years since the Yale Conference. Therefore, there is a need to incorporate these developments into our Charter. In addition, from discussions with our ISBM members, we have learned that there is a need for clarification on how we as a society define behavioral medicine. As ISBM is a large and diverse international organization, with varying perspectives on behavioral medicine among our members, it is important to consider these various viewpoints on how we define our field.

The ISBM Charter defines behavioral medicine as follows: “Behavioral medicine can be defined as the interdisciplinary field concerned with the development and integration of psychosocial, behavioral, and biomedical knowledge relevant to health and illness and the application of this knowledge to prevention, etiology, diagnosis, treatment, and rehabilitation. The scope of “behavioral medicine” extends from research efforts to understand fundamental biobehavioral mechanisms; to clinical diagnosis and intervention; to disease prevention and health promotion” [3]. Although still remarkably adequate, various components of the definition need to be addressed and updated to better align with new developments in the field and the views of our members. We will point out these issues and then make a proposal for an updated definition of behavioral medicine in our Charter.

## Scope

There is a need to update and clarify the scope of behavioral medicine. The term *biobehavioral mechanisms* refers to the interaction of biomedical processes (i.e., biological and disease-related processes) with a broad range of other processes, including psychological, social, societal, cultural, and environmental factors that contribute to health and disease. “Bio-” refers to biological and disease-related processes, while “behavioral” refers to psychological, social, societal, cultural, and environmental factors that contribute to health and disease [4, 5].

An area in need of clarification is that there is a tendency to equate and limit the definition of biobehavioral mechanisms to primarily biomedical-oriented processes: the interaction of behavior with biological and disease-related systems, such as the immune system, autonomous nervous system, endocrine system, central nervous system, genetics, metabolism, or disease activity [6]. A specific example is the impact of stress on the immune system. Although an understanding of these mechanisms is of vital importance and part of biobehavioral research, the role of psychological and social mechanisms in health and disease is well established. Research on biobehavioral mechanisms may focus on psychological and social mechanisms related to health and disease, such as conditioning of somatic responses, cognitive and behavioral coping styles, the role of emotions and behavior, the acquisition and maintenance of health behaviors, and the influence of family and partner. A specific example is the impact of depressed mood on health behaviors in patients with a somatic disease. Similarly, societal, cultural, and environmental mechanisms have been identified, including the impact of socioeconomic status, neighborhood, (early) adversity, and work on health and disease. A specific example is the exposure to multiple risk factors as the explanatory mechanism for the socioeconomic status-health gradient. There is a need to clarify in the Charter that biobehavioral mechanisms refer not only to the interaction of behavior with biological and disease-related systems, but also to a broad range of processes in the psychological, social, societal, cultural, and environmental domain that contribute to health and disease; this may or may not include attention to specific biological and disease-related systems.

The description of the area of *clinical diagnosis and intervention* does not seem to require an update. However, it is important to reiterate that in behavioral medicine, work specific to clinical diagnosis and intervention occurs in the context of physical health processes and outcome. Behavioral medicine needs to be distinguished from mental health (see below).

In the area of *disease prevention and health promotion*, major developments have taken place. The role of health policy, health promotion, and health services as determinants of health and disease has been emphasized. Health policy (with regard to smoking or vaccination, for example), health promotion (e.g., local initiatives enabling people to increase their level of physical activity), and health care services (e.g., the availability of primary care services) have a strong impact on health and disease. We suggest to use *public health* to refer to this area. WHO [7] defines public health as follows: “Public health refers to all organized measures (whether public or private) to prevent disease, promote health, and prolong life among the population as a whole. Its activities aim to provide conditions in which people can be healthy and focus on entire populations, not on individual patients or diseases.” This definition seems to encompass the recent activities and developments in this area of behavioral medicine.

## Research

There is a need to clarify that research is an essential characteristic of all three areas of behavioral medicine, i.e., biobehavioral mechanisms, clinical diagnosis and intervention, and public health (disease prevention and health promotion). The charter is sometimes misunderstood as limiting research to the area of biobehavioral mechanisms only. Clearly, this is not the case: behavioral medicine research as defined in our Charter was intended to imply that research is a fundamental characteristic of all areas of behavioral medicine. Therefore, behavioral medicine research is considered a broad field of investigation that not only includes research on biobehavioral mechanisms, but also on clinical diagnosis and intervention, and public health.

## Application

With regard to application, behavioral medicine has seen a growing emphasis on the development and evaluation of implementation strategies. The implementation strategies concern the clinical setting as well as the public health setting, with an emphasis on health promotion. The Charter currently lists application of knowledge to *prevention*, *etiology*, *diagnosis*, *treatment*, *and rehabilitation*. In order to take the recent developments into account, we propose to add *health promotion* to this list. Further, there is a need to delete “etiology” from this list. Prevention, health promotion, diagnosis, treatment, and rehabilitation all concern interventions. Etiology is in a different category, related to causal mechanisms, and does not fit here. Finally, there is a need to add care: care is different from diagnosis, treatment, and rehabilitation. These considerations result in mentioning prevention, health promotion, diagnosis, treatment, rehabilitation, and care.

## Multidisciplinary Field

Various terms have been used to refer to the involvement of multiple disciplines in health research and practice. Choi and Pak [8] use the terms multidisciplinarity, interdisciplinarity, and transdisciplinarity to refer to a continuum of increasing involvement of multiple disciplines. They suggest the more general term “multiple disciplinary” for when the nature of involvement of multiple disciplines is unspecified. The involvement of various disciplines is a defining characteristic of behavioral medicine, but the strength of the involvement of various disciplines may vary. Although this would suggest “multiple disciplinary” as the most appropriate term, we prefer the more traditional term “multidisciplinary” to define behavioral medicine.

## Related Fields

Perhaps most difficult, there is a need to clarify the relationship and distinction between behavioral medicine, mental health, health psychology, and psychosomatic medicine. The exact definition and positioning of behavioral medicine varies among our member societies, leading to discussions on the nature of behavioral medicine.

We suggest that behavioral medicine and mental health should be seen as different fields. Mental health is primarily concerned with mental disorders such as schizophrenia, anxiety disorder, and major depressive disorder. Somatic diseases or symptoms may contribute to mental disorders (e.g., an increased incidence of depression after the diagnosis of cancer). In addition, there is the possibility of common causal pathways of somatic and mental disease (e.g., inflammation might play a role in both somatic and mental disease). These are areas of overlap between mental health and behavioral medicine. Functional syndromes or medically unexplained symptoms constitute another area of overlap. Further, knowledge obtained in one field may be applied in the other (e.g., knowledge obtained in the field of mental health on how to treat depression can be applied in the treatment of patients with cancer and depression). Nevertheless, despite overlap and exchange, behavioral medicine and mental health are different fields. Mental health primarily concerns mental disorders, while the focus of behavioral medicine is primarily on the role of behavior in somatic disease and symptoms.

Overlap and exchange are also characteristics of the relationship between health psychology and behavioral medicine. However, multidisciplinary membership is a defining characteristic of behavioral medicine, while health psychology is a primarily mono-disciplinary field [9]. The ISBM Charter and Bylaws emphasize and require multidisciplinary membership: ISBM members have a background in Medicine, Psychology, Public Health, Sociology, Nursing, Nutrition, Physiotherapy, and other disciplines. Behavioral medicine provides an exciting opportunity for cross-fertilization and exchange of ideas between scientists working in different disciplines. Health psychology, as defined by APA Division 38, focuses on ‘advancing contributions of the psychology discipline toward understanding health and illness through basic and clinical research and by encouraging the integration of biomedical information about health and illness with current psychological knowledge’ [10] Clearly, this implies multidisciplinarity, but the emphasis is on psychology; in this respect, health psychology differs from behavioral medicine, which involves a multidisciplinary approach as a defining characteristic.

Psychosomatic medicine primary focuses on “psychosocial factors affecting individual vulnerability and course and outcome of any type of disease; and the integration of psychological therapies in the prevention, treatment and rehabilitation of medical disease (psychological medicine)” [11]. Psychosomatic medicine differs from behavioral medicine by a more exclusive and in-depth focus on the medical aspects of the interaction between psychosocial factors and disease, and the implementation of this approach in clinical care; public health and health promotion are not the primary focus.

We argue that fundamental differences exist between behavioral medicine, mental health, and health psychology, while psychosomatic medicine is in the same field but has a more in-depth focus on medical processes. While the intent is not to define other fields and it might not be appropriate to address the relationship with other fields in the ISBM Charter, there is a need to distinguish behavioral medicine from other disciplines and document such distinctions in ISBM.

## Definition and Scope of Behavioral Medicine

**Fig. 1:**
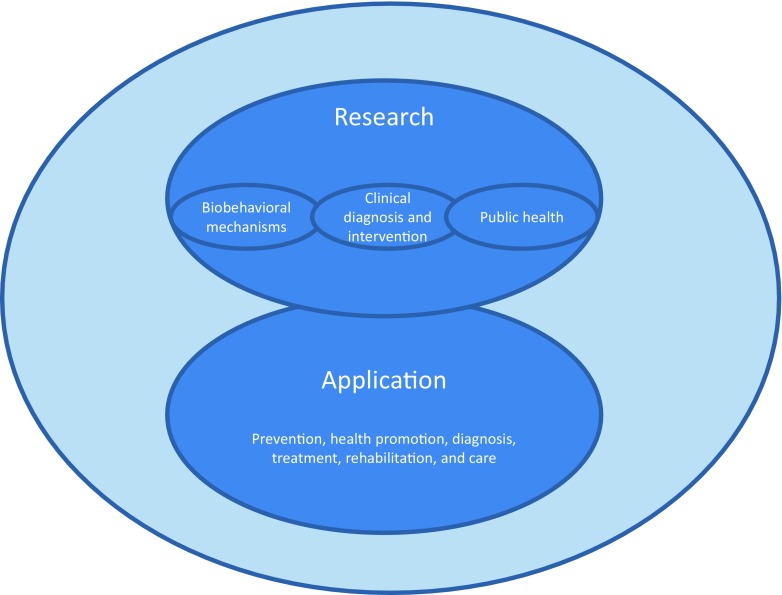
The definition and scope of behavioral medicine

These considerations lead to the following proposal on the definition and scope of behavioral medicine in the ISBM Charter: *Behavioral medicine can be defined as the multidisciplinary field concerned with the development and integration of biomedical and behavioral knowledge relevant to health and disease, and the application of this knowledge to prevention, health promotion, diagnosis, treatment, rehabilitation, and care. The scope of behavioral medicine extends from biobehavioral mechanisms (i.e., the interaction of biomedical processes with psychological, social, societal, cultural, and environmental processes), to clinical diagnosis and intervention, and to public health.* This definition is illustrated in Fig. 1.
